# Advancing a collective vision for equity‐based cocreation through prototyping at an international forum

**DOI:** 10.1111/hex.14041

**Published:** 2024-04-03

**Authors:** Michelle Phoenix, Sandra Moll, Alexa Vrzovski, Le‐Tien Bhaskar, Samantha Micsinszki, Emma Bruce, Lulwama Mulalu, Puspita Hossain, Bonnie Freeman, Gillian Mulvale, CoPro Consortium

**Affiliations:** ^1^ School of Rehabilitation Sciences, CanChild McMaster University Hamilton Ontario Canada; ^2^ School of Rehabilitation Sciences McMaster University Hamilton Ontario Canada; ^3^ Ahousaht First Nation Ahousat British Colombia Canada; ^4^ Indigenous Project Coordinator, School of Medicine Toronto Metropolitan University Toronto Ontario Canada; ^5^ Health Research Methods, Evidence, and Impact (Health Policy) McMaster University Hamilton Ontario Canada; ^6^ Global Health McMaster University Hamilton Ontario Canada; ^7^ School of Social Work and Indigenous Studies Department McMaster University; Algonquin/Mohawk, Member of the Six Nations of the Grand River Canada; ^8^ Health Policy and Management, DeGroote School of Business McMaster University Hamilton Ontario Canada; ^9^ McMaster University Hamilton Canada

**Keywords:** arts‐based research, caregiver engagement, cocreation, codesign, equity, integration, patient engagement

## Abstract

**Background:**

Cocreation has the potential to engage people with lived and living experiences in the design and evaluation of health and social services. However, guidance is needed to better include people from equity‐deserving groups (EDGs), who are more likely to face barriers to participation, experience ongoing or historical harm, and benefit from accessible methods of engagement.

**Objective:**

The aim of this international forum (CoPro2022) was to advance a collective vision for equity‐based cocreation.

**Design:**

A participatory process of engagement in experiential colearning and arts‐based creative and reflective dialogue. Visual prototypes were created and synthesised to generate a collective vision for inclusive equity‐based cocreation.

**Setting and Participants:**

The Forum was held at the Gathering Place by the Grand River in Ohsweken, Ontario, Canada. A total of 48 participants attended the forum. They were purposely invited and have intersecting positionalities (21 academic experts, six experience experts, 10 trainees, and 11 members of EDGs) from nine countries (Bangladesh, Botswana, Canada, England, Italy, Norway, Scotland, Singapore, Sweden).

**CoPro2022 Activities:**

CoPro2022 was an immersive experience hosted on Indigenous land that encouraged continuous participant reflection on their own worldviews and those of others as participants openly discussed the challenges and opportunities with engaging EDGs in cocreation activities. Visual prototypes and descriptions created in small groups were informed by participants' reflections on the panel presentations at the Forum and their own experiences with equity‐based cocreation. Following the event, the authorship team inductively coded themes from the prototype descriptions and met to discuss the cross‐cutting themes. These informed the design of an illustrated collective vision for Equity Based Co‐Creation (EqCC).

**Results:**

Six prototypes were cocreated by each small group to illustrate their vision for EqCC. Within these, four cross‐cutting themes were identified: (i) go to where people are, (ii) nurture relationships and creativity, (iii) reflect, replenish and grow, (iv) and promote thriving and transformation. These four themes are captured in the Collective EqCC Vision to guide a new era of inclusive excellence in cocreation activities.

**Patient or Public Contribution:**

Service users, caregivers, and people with lived experience were involved in leading the design of the CoPro2022 and co‐led the event. This included activities at the event such as presenting, facilitating small and large group discussion, leading art‐based activities, and reflecting with the team on the lessons learned. People with lived experience were involved in the analysis and knowledge sharing from this event. Several members of the research team (students and researchers) also identified as members of EDGs and were invited to draw from their personal and academic knowledge.

## 1 INTRODUCTION

Terms such as cocreation, codesign, and coproduction have been used to refer to knowledge generation projects in which people with lived and living experience are respected as knowledge experts who can contribute beyond a participant role to inform the research process, using creative methods and relational processes, tailored in local contexts, to affect change in systems and governance.^1^ These approaches require collaboration, partnership, and power sharing between people with lived and living experience and people who hold other linked roles, such as clinicians, organisational leadership, policy makers, and researchers.^2^, ^3^ Cocreation has been applied in various disciplines, such as business, social science, computer science, design and engineering, with widespread application in health and social care, technology, and policy.^1^, ^4^ The widespread and rapid uptake of these approaches (which we will collectively refer to as cocreation) has raised questions about the key tenets of cocreation, the value added by cocreation approaches, and the potential harms in cocreation projects.^3^, ^5^

While there is variation in participatory approaches to research, we recognise core principles of cocreation as: ‘distribution of power in research, amelioration of the human experience and positive societal impact’,^3^ ^,p.1^ as necessary components that underlie the cocreation philosophy and methods. More specifically, researchers who engage in cocreation projects are invited to critically examine their own positionalities and worldviews, meaningfully include people with lived and living experience in projects alongside service providers and researchers, and integrate methods that are arts‐based, flexible, iterative and dynamic.^3^ It is expected to produce research that moves quickly from development to implementation,^1^ decrease research waste by focussing primarily on the priorities of service users and care providers,^6^ and promote a democratic and innovative approach to addressing complex health problems and policy development.^4^ The emphasis on interpersonal relationships and active collaboration in cocreation allows for avenues of power redistribution,^7^ catalysing radical forms of empathy,^8^ and the development of trust.^9^ This may be contrasted with traditional forms of research in which participants share their knowledge via data collection (e.g., interviews or focus groups) and the researchers maintain the power to interpret or analyse the data, share the findings, and make changes to the interventions or policies.

Cocreation has been used to engage various populations who face systemic oppression that limits their inclusion in the design, use, or evaluation of health and social services. These populations have been referred to as vulnerable or marginalised, however this language is contested as it may perpetuate vulnerability as an individual trait, whereas societal processes that perpetuate historical legacies and (re)‐produce structural precarity must be considered.^10^, ^11^ We use the term equity‐deserving groups (EDGs) to refer to populations of varying sociohistoric, political, economic and cultural contexts, both locally and globally, who are and have been more likely to experience systemic oppression via population‐level barriers to health and well‐being and limitations in access to services (e.g., groups that include Black, Indigenous, racialized, disabled, 2SLGBTQI+ people). Cocreation has been used with diverse populations to develop a range of health and social services and tools, for example, mental health services with youth,^12^ research training tools with youth with disabilities and caregivers,^13^ transit systems with older newcomers,^14^ and health promotion activities with older adults.^15^ A common element in these diverse projects is the inclusion of people with lived and living experience. The language for people who develop expertise through their own experience (e.g., with a health condition or use of a service) has been debated,^16^, ^17^, ^18^ however we have used the term experience expert (EE) to recognise the valuable insights and valid embodied knowledge(s) rooted in body consciousness that people have and may share if provided with opportunities to do so. A frequently raised criticism in cocreation research is a tendency to engage people with lived and living experience that have the most privilege.^19^ Intersectionality theory, originally described by Kimberlé Crenshaw in 1989, can be usefully applied to consider which aspects of our interconnected identities may be afforded the most unearned privilege or increase the likelihood of experiencing systemic oppression. People are multidimensional and therefore have multiple identities that either bestow or deny certain privileges, social currencies and cultural capital; as such the dynamic interactions of multiple systemic and structural inequalities must be considered. For example, cocreated research broadly has typically engaged white, English‐speaking patients or carers (typically mothers), who have the financial means, language and literacy skills to engage in research.^3^, ^20^, ^21^ This may include ‘super patients’ who are frequently engaged in research projects and have been socialised to the research process, raising questions about whether their experience is typical of service users and potentially overrepresented in cocreated services and research.^3^, ^19^ Limiting opportunities for engagement to those who are familiar with the team or privileged risks absolving researchers of their responsibility to address the needs and priorities of EDGs and may reproduce the inequalities that exist in health and social systems in cocreation processes and outcomes.^5^, ^22^, ^23^ Therefore, evidence is needed to guide the philosophy and methods that can promote equity in the cocreation of health and social services.

In 2017, members of this authorship team held a 2‐day International Symposium with researchers and EEs to explore challenges, opportunities, and solutions to cocreating health and other public services with vulnerable and disadvantaged populations. This event led to advancements in the practices of cocreation with vulnerable populations,^9^ the methods of creative elicitation in cocreation^12^ and guidance about relationship creation and navigation.^7^ However, a need was identified for future work to focus explicitly on equity in cocreation by increasing recognition and inclusion of EDGs, to embed immersive and experiential learning, and to create a vision for systemic, collective, and transformative change. Therefore, between May and October 2022, a series of online and in‐person knowledge mobilisation events (the CoPro2022 series) was held to gather international researchers and EEs to create a shared vision for inclusive excellence in Equity‐Based Co‐Creation (EqCC) through collaborative arts‐based dialogue and reflection. The seminal event in this series was the CoPro2022 Forum.

## 2 THE CoPro FORUM

The CoPro2022 Forum was a 3 day in‐person international forum (the Forum) held in person at the Gathering Place at Six Nations of the Grand River Territory in Ontario, Canada in August, 2022. For more information, see our website (https://codesign.mcmaster.ca/copro-2022/copro-2022-3-day-symposium/).^24^ The forum aimed to share knowledge and experiences to create a collective vision for EqCC. The structure, principles, and theoretical foundations of the CoPro2022 series are described elsewhere.^25^ The focus here is to present the vision for EqCC via the visual prototypes that were created at the forum.

### 2.1 Participants

People were invited to the forum if they were known to members of the organising committee for having a background and expertise in cocreation activities such as codesign, coproduction, and collaborative, community‐based participatory approaches involving EDGs. There was intentional inclusion of representatives from EDGs where possible. The organising committee was composed of members of McMaster's Co‐Design Hub (aimed at advancing codesign with structurally vulnerable groups^26^ and colleagues who crossed multiple roles for example, as EEs (e.g., as international students or people with experience with health service use or as family/carers), interdisciplinary students and researchers (e.g., from rehabilitation science, business, social science), and as members of EDGs. The organising committee considered the following criteria when determining who to invite: (1) Considering whether the individual had enough experience with cocreation with EDGs to meaningfully contribute to the reflection and dialogue, (2) diversity in health and social care contexts and populations included, (3) geographic diversity, and (4) balance of people with diverse roles (e.g., EEs and members from diverse EDGs, trainees, interdisciplinary researchers).

A total of 48 purposely invited participants with intersecting positionalities (21 academic experts, six EEs, 10 trainees, and 11 members of EDGs) from nine countries (Bangladesh, Botswana, Canada, England, Italy, Norway, Scotland, Singapore, Sweden) attended the Forum. The intention was to keep the size manageable so that everyone could meaningfully participate and to remove financial barriers to access for EEs by covering all travel and accommodation costs. This approach may have limited the inclusion of more people who were unable to travel or commit to a 3 day in‐person event. Online events were held in May–October as part of the CoPro2022 series, while data was not formally collected at those events, key themes were captured through artistic and poetic summaries that were brought forward during discussions at the in‐person event so as to further inform the vision of equity‐based cocreation reported here. All small group discussions took place in groups of 4–6 participants and 1–2 facilitators that intentionally included people who held different social positions and roles.

### 2.2 CoPro2022 forum activities

In preparation for the forum, participants were provided with background information about the event activities and suggested reflection questions. Participants were asked to consider their starting point, in terms of the attitudes they would bring to the cocreation activities and forum discussions. Questions from McMaster Co‐Design Hub's published reflexive questions were precirculated to prompt reflection on people's values and assumptions, openness to learning and compassion, interrogation of power, and navigation of time and resource constraints to prioritise dialogue and cocreation.^3^

The forum was designed to include opportunities for relationship building (e.g., paddling, shared meals), arts‐based experiential and cultural activities led by community members (e.g., Indigenous dancing and beading), and a mix of panel presentations with facilitated group discussions that embedded creative methods.^27^ The purpose of relationship building was to promote feelings of inclusion, create an environment where vulnerability was welcomed when sharing knowledge and ideas, and to serve as a foundation for future collaboration. Therefore, we were not prescriptive in determining who should participate together in the activities or small groups and instead offered choice and encouraged new relationships.

The first 2 days featured interactive panel discussions comprised of short presentations about the challenges and opportunities in engaging EDGs in cocreation (agenda can be viewed here: https://codesign.mcmaster.ca/copro-2022/copro-2022-3-day-symposium/). Day 1 panellists discussed cocreation in Black, Asian and Minority Ethnic, immigrant and refugee, end of life, and humanitarian contexts. At the end of this panel, participants worked in small groups to identify important touch points (emotionally charged memorable moments in experiences) from challenges and opportunities in cocreating with EDGs.^2^, ^12^ Participants selected their own small groups and they were invited to select tables with people they had not met before and to ensure that tables included a mix of EEs, members of EDGs, trainees, and researchers.

Day 2 morning panellists presented successful case study examples of ongoing cocreation models to inspire thinking about what was working well and what could be enhanced. Following the presentations, participants worked in six small groups to cocreate a visual prototype of their vision for EqCC. Again, the groups were not predetermined, and diversity in membership and forming new groups was encouraged to bring forward new ideas. Prototypes are commonly used in cocreation to ‘evoke a focused discussion in a team, because the phenomenon is “on the table”’,^28^ ^,p.6^ while examining and addressing real‐world problems. Facilitator notes and the visual prototypes were used to capture the discussion at the forum. Each small group then verbally shared their prototype with the full plenary group. Facilitators from our team used strategies that promoted involvement from all participants (e.g., providing opportunities for written contributions on sticky notes, encouraging all participants a chance to ‘hold the pen’ when prototyping on the white board, inviting oral contributions from all group members, asking for a volunteer to share a verbal summary back with the full group). Facilitators observed that the discussion was balanced among all group members and people from various roles took the opportunity to share back with the large group. A challenge experienced was fully including a participant who did not speak English; while we had translation support available, we had to be mindful of slowing the conversation to make space for understanding and contributions. While this paper focuses on the lessons learned from the group discussions and creative products produced following the panel presentations, it is important to appreciate the relational and experiential context in which the collective vision was developed.

### 2.3 Synthesising and sharing the prototype meanings

Following the event, facilitators from each small group prepared a summary of their prototype description in text. These summaries were shared back with the CoPro2022 participants in each group to check for accuracy. All authors reviewed the original six prototypes and written descriptions, then met to discuss the key points from each image and how they related to one another. L. B. created a digital version of each prototype. M. P. inductively coded the descriptive summaries of the six prototypes to identify key elements of EqCC within each. These key elements were compared across the prototypes to identify common and unique elements that arose across the prototypes. Four members of the authorship team members (A. V., G. M., S. M., M. P.) reviewed the coded text and met to discuss to key elements of EqCC and generated four cross‐cutting themes that synthesised these elements. This group then met with a digital artist to refine the original six prototypes and to develop a ‘meta image’ representing a Collective EqCC Vision drawn from across the individual prototypes. This process allowed the team to maintain the rich visual elements that were included in the original protypes, while expanding on the shared meaning that emerged from across the small group discussions.

## 3 LESSONS LEARNED

**Figure 1:**
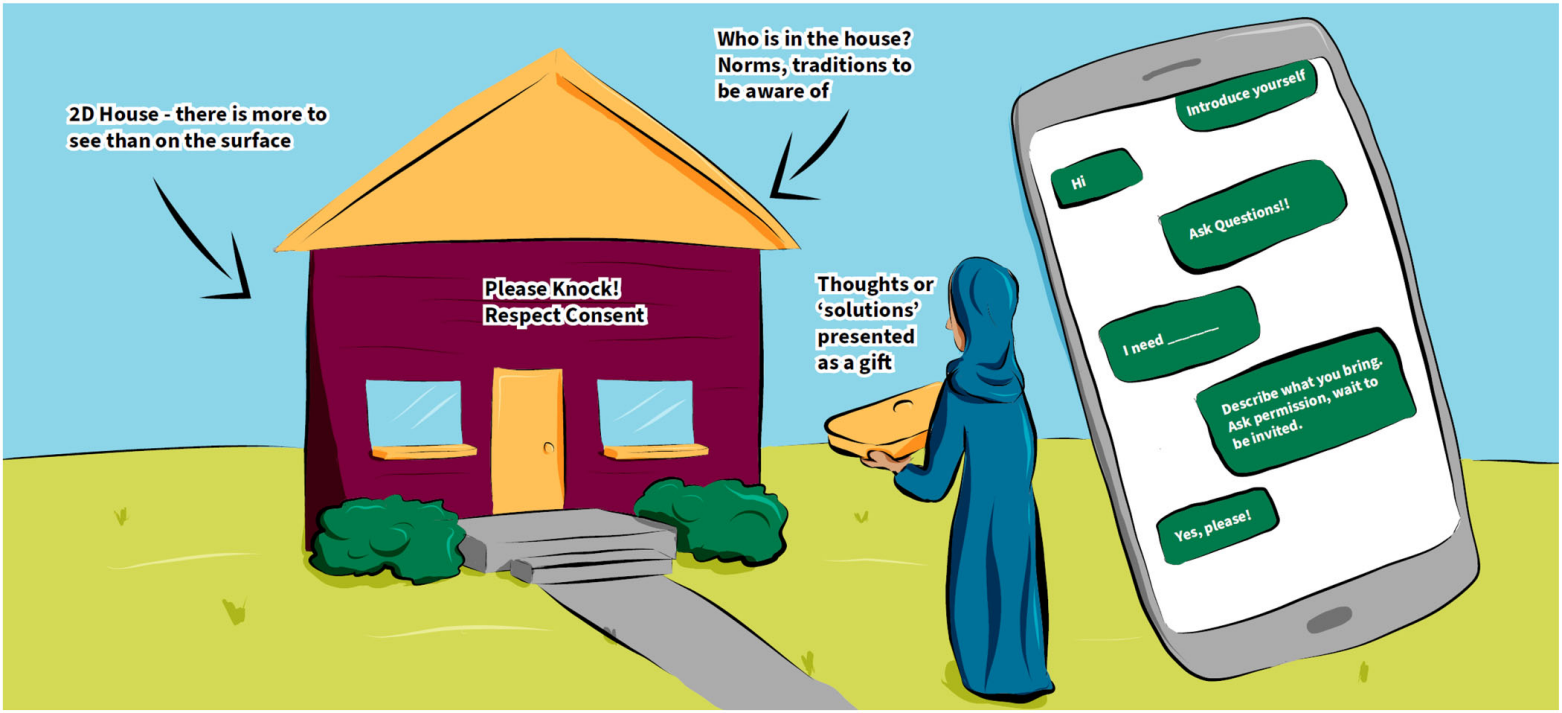
Two dimensional house.

**Figure 2:**
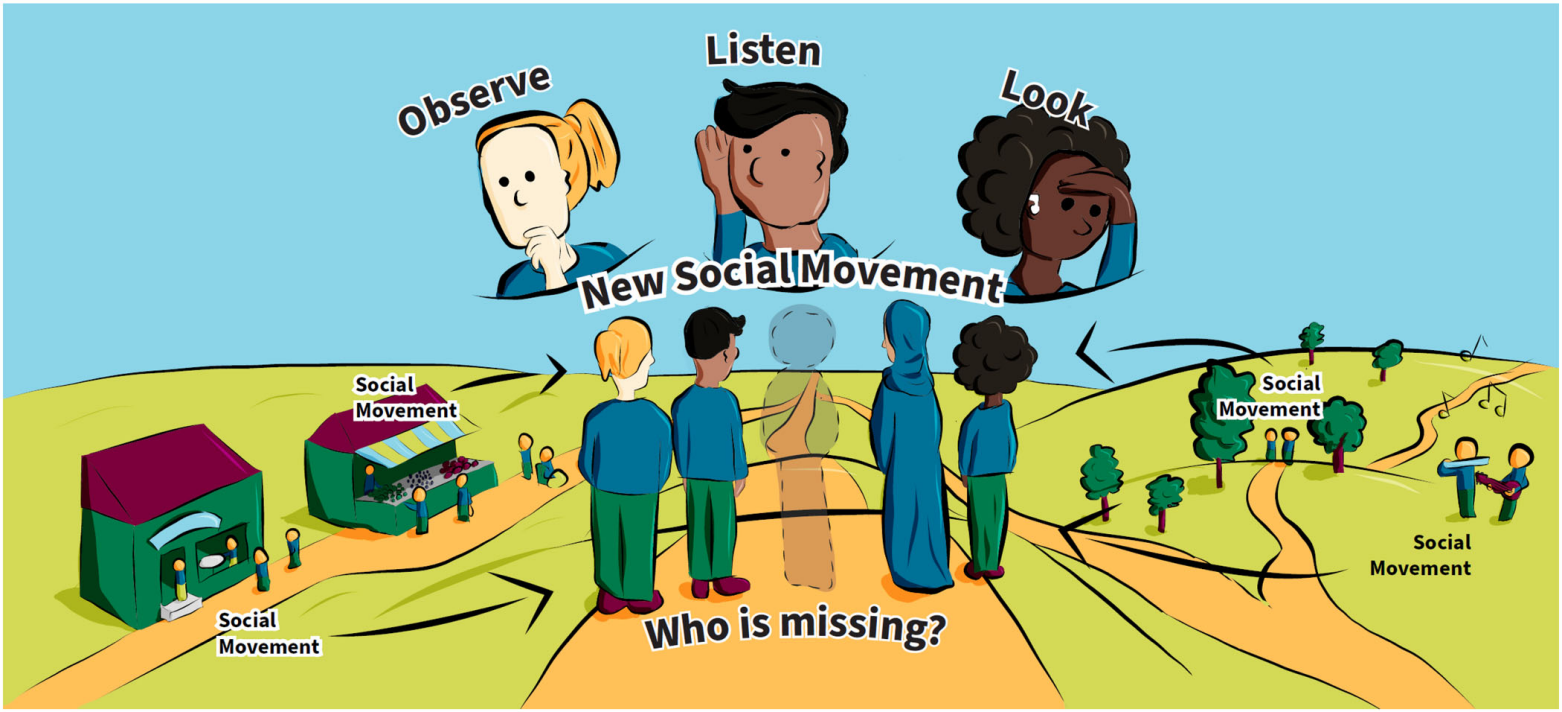
The people's social movement.

**Figure 3:**
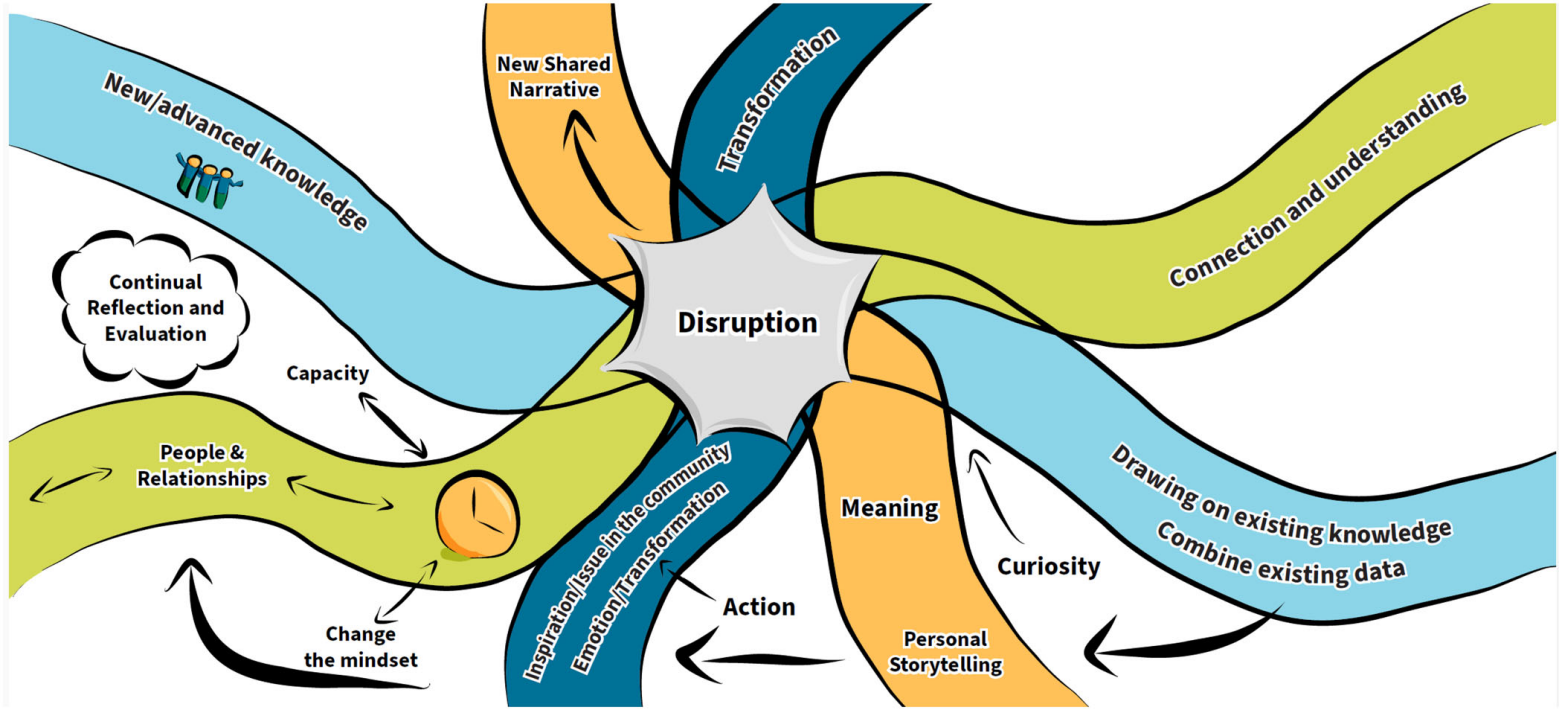
Disruptive pathways.

**Figure 4:**
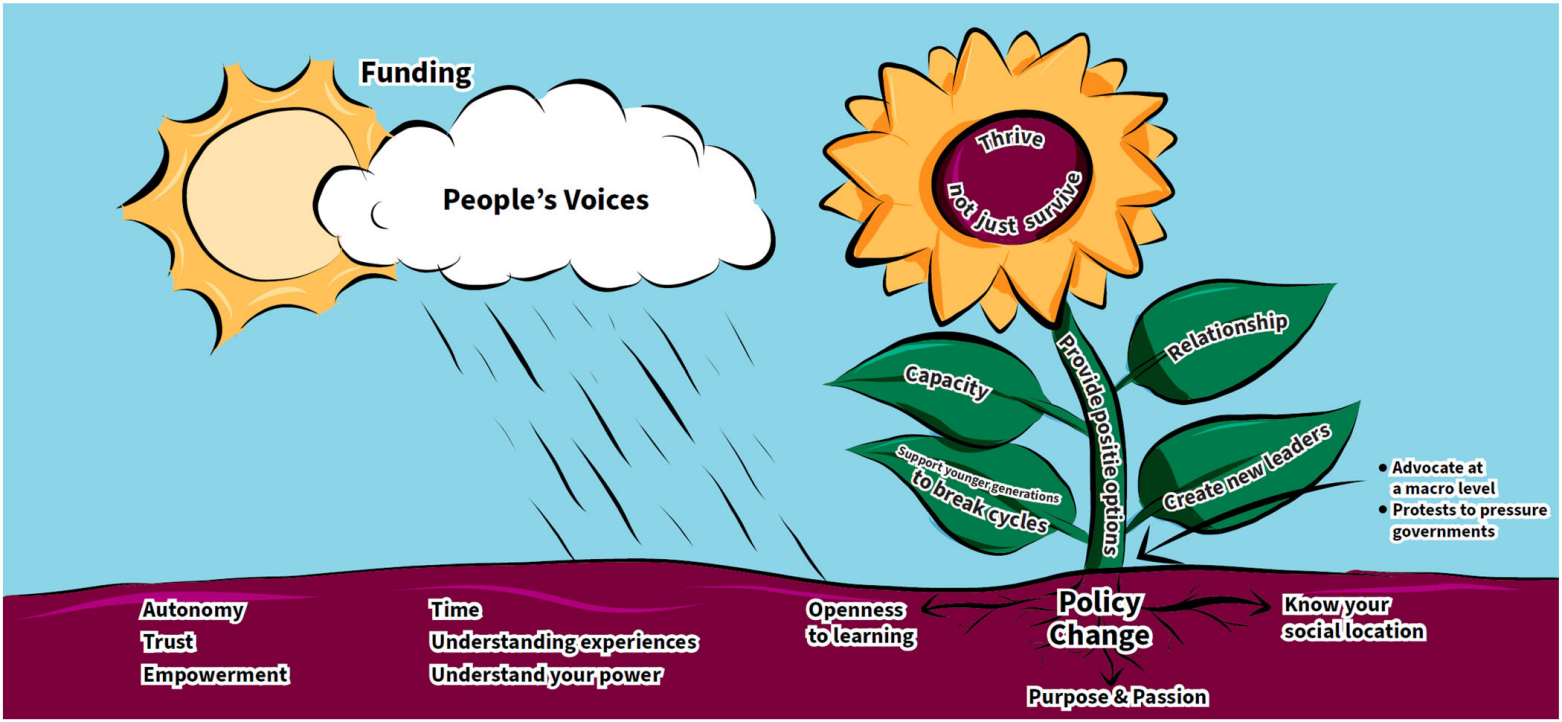
Sunflower.

**Figure 5:**
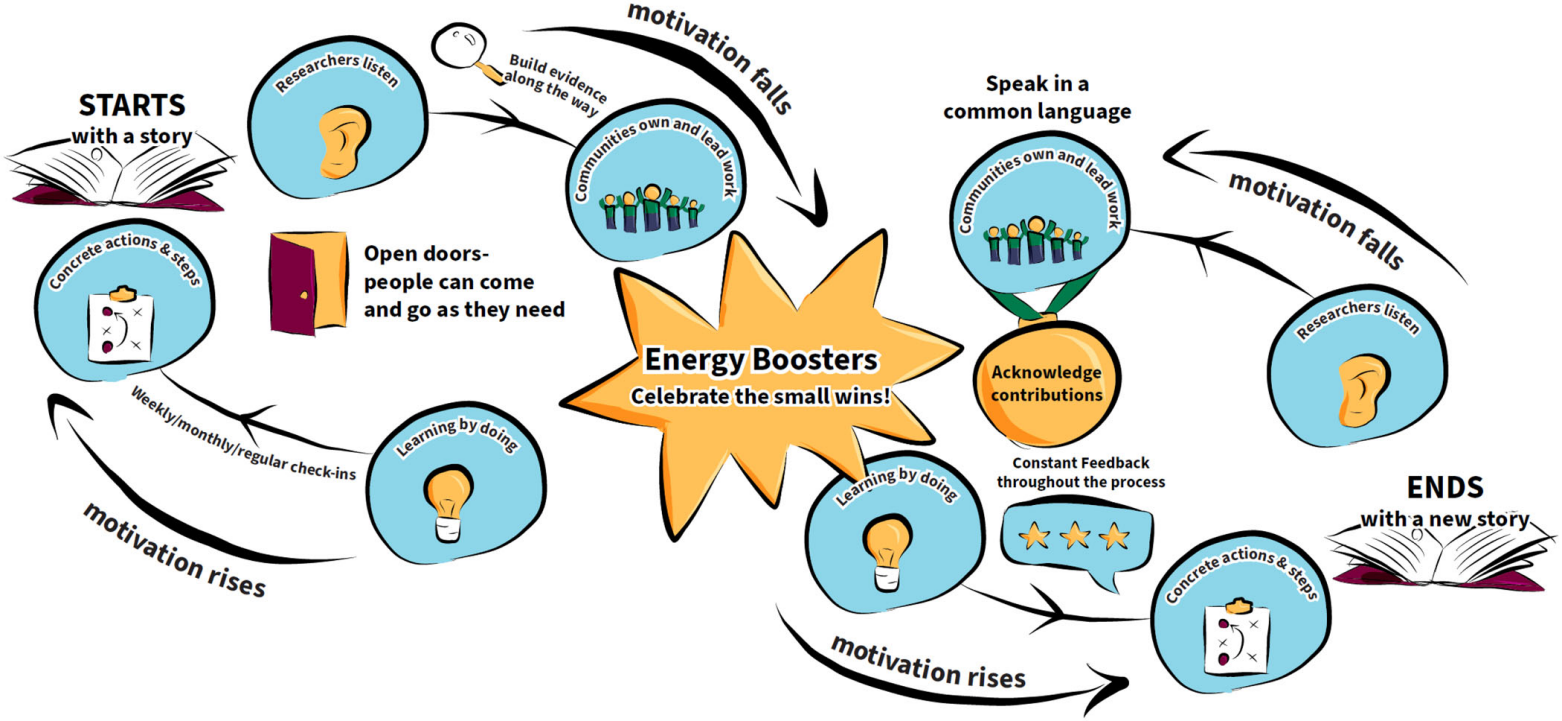
The infinity loop.

**Figure 6:**
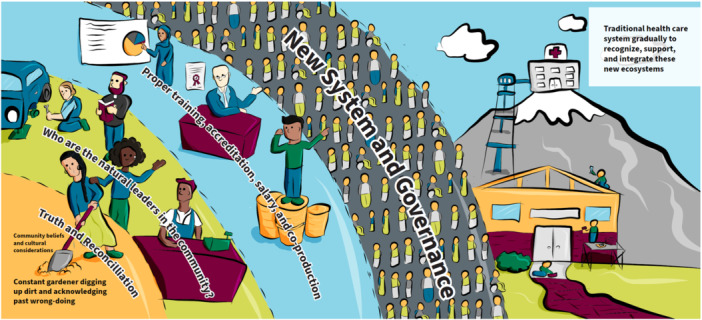
New community ecosystem.

**Figure 7:**
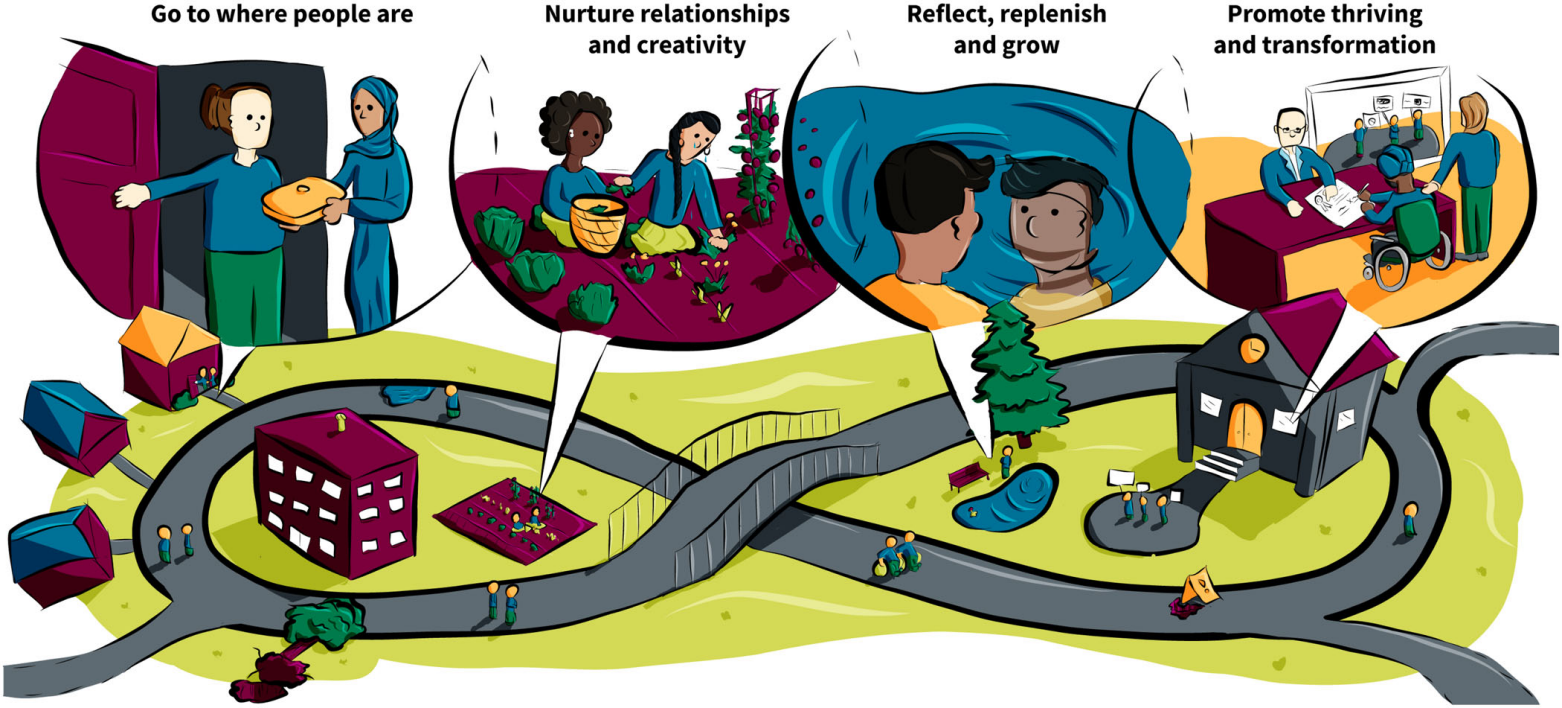
A collective vision for equity‐based cocreation.

The lessons learned are presented in two parts. Part A provides an overview and description of the six prototype images presented in Figures 1, 2, 3, 4, 5, 6. Part B presents the Collective EqCC Vision (Figure 7), which embeds and describes the four EqCC cross‐cutting themes: (1) Go to where people are; (2) Nurture relationships and creativity; (3) Reflect, replenish and grow; and (4) Promote thriving and transformation.

### 3.1 Part A: Overview and description of the forum prototypes

The first prototype (Figure 1) pertains to the *engagement phase* of EqCC. A house, representing the community, was drawn as a two‐dimensional, flat and generic shape, which is all that might be seen if people don't look deeply. In reality, houses are three‐dimensional with more depth, nuance, and complexity than can be seen at face value. To avoid missing this multidimensionality, cocreators must include multiple perspectives shared via storytelling, and honour different worldviews. Cocreators must figuratively knock, announce themselves, share their purposes, identity, and values to build trust before working together. Cocreators must recognise that there are communal and familial structures within communities and they must take time to develop cultural humility by following the rules of the community (e.g., gifts, cultural greetings, dress code) rather than relying on their assumptions.

The second prototype (Figure 2) addresses the question *Who is missing?* from existing processes and why. Novel engagement methods are encouraged including spending time in community venues such as gardens, pubs, book club meetings to build relationships with community members from EDGs and create networks with others sharing similar values, interests, and ideas. Cultural activities (e.g., music, dancing) that resonate with different communities should be incorporated in EqCC processes so that community members feel honoured, interested and included. As networks grow over time, they act as social movements that can shift power to better honour the contributions of EEs from EDGs.

This prototype illustrates the *transformative power* of EqCC processes through four intersecting pathways on a journey of personal, relational, epistemic and action‐oriented transformation. The personal pathway focuses on sharing stories to develop new shared meanings and narratives. The relational pathway involves relationship‐building through creating connection and mutual understanding by challenging rigid mindsets, building new capacity to challenge traditional power structures. The epistemic pathway draws upon and honours different types of knowledge (e.g., research, experiential and cultural) to advance mutual understanding to foster new discourses. The action‐based pathway engages people at an emotional level to inspire transformation to address issues identified by communities. Curiosity, reflection and evaluation drives work along all pathways, with a continuous feedback process that permits participants to take risks, make ‘mistakes’, learn from each other, and then recalibrate, re‐engage and make course corrections when needed. The vision suggests that when the paths intersect, there is the potential for disruptive change.

The fourth prototype (Figure 4), has a sunflower as its focal point, with a vision of a *nurturing garden* wherein EDGs will ‘thrive not just survive’. The roots of this growth can be found in openness to learning, purpose and passion, and understanding our own social locations. Policy change is illustrated as being central to facilitate growth, in a context of autonomy, trust, empowerment, time, understanding experiences and personal power. Funding and listening to people's voices provide the energy for growth (sun and rain images), through mechanisms such as capacity and relationship building, supporting younger generations, and creating new leaders.

This prototype envisions EqCC as a *fluid and continuous process* owned and lead by communities that starts with sharing stories of lived experience and ends with the cocreation of new stories that resonate with communities. There is recognition that telling and listening to stories of lived experience requires tremendous energy by both the storyteller and the listeners. The vision also suggests that researchers need to create conducive processes for listening to and honouring lived experience, enabling EEs to take space for self‐care by leaving and re‐entering processes as needed. There is also recognition that transformative change takes time, and there will be periods where motivation waxes and wanes. Celebrating small wins and acknowledging people's contributions will help to boost motivation. Constant critical reflection at planned checkpoints can ensure progress is being made and concrete steps taken to operationalise the ideas being generated. The work does not stop with the end of a project but requires continuous efforts.

The final prototype (Figure 6) presents a vision of how community‐based cocreation processes can *build community ecosystems* that gradually dismantle oppressive elements within existing healthcare systems (shown in the top right as a ‘lofty peak’ initially removed from the community) and lead to a new transformed and integrated system. Transformation begins within communities, as shown in the bottom left corner and moves outward to the right, first by working in a sustained way with members of a community to build understanding and trust, by uncovering harms, offering apology and efforts toward reconciliation, and embracing cultural knowledge and traditions. Next, natural leaders in the community are identified who are well‐poised to notice concerns, be sounding boards, and trusted links to more formal health and social services when needed. These leaders may hold formal (e.g., civic and religious) or informal (e.g., taxi drivers, hairdressers, baristas) roles. Over time, the essential work of these natural leaders is recognised by the formal system through training, salary and accreditation and they become a new community‐based culturally safe, front‐line health workforce. Through mutual learning, the existing system is transformed to become more welcoming and culturally safe of EDG community members, and the ecosystem's role in providing services will be recognised and maintained through new cogovernance structures.

### 3.2 Part B: Collective vision for EqCC and the four EqCC cross‐cutting themes

Figure 7 presents a *collective vision for EqCC* based on cross‐cutting themes captured in the six prototypes. As shown in the image, there is an infinity loop pathway at the centre that is designed to illustrate how EqCC is dynamic, continuous, and rooted in a community ecosystem that can create a grass‐roots social movement for transformation. The infinity loop illustrates individuals and groups of people who are embedded in distinct, but connected, micro and macro environments, and have opportunities to come together, leave the path and return. There are barriers along the path representing the mistakes, harms, and pitfalls that frequently occur in cocreation research, necessitating the constant need for reflexivity, feedback, and ongoing learning. The infinity loop reminds us that it may take time for change to happen, which requires sustained momentum and accountability. The ‘call‐out’ images along the pathway represent the four cross‐cutting themes or processes embedded in equity‐based cocreation. These elements represent imperatives in enacting cocreation processes that are inclusive and accessible to those who may face a range of structural inequities. Ultimately, relationships, skills, and knowledge built in one environment can be galvanised to create change in new environments, building towards systemic change in structures, policies, governance.

### 3.3 Theme 1: Go to where people are

The first theme ‘go to where people are’ is based on a foundational understanding that EqCC should begin once there is alignment between the readiness of the people who will share their stories and the community in which the cocreation will be completed; the researcher needs to understand and respect the readiness of individuals and communities. As illustrated in the roots of the sunflower (Figure 4), researchers must enter the process with an openness to learning and an awareness of their own social location and power. The image of the house (Figure 1) expands on the idea that researchers need to enter with humility, to set aside their own assumptions, and to honour and respect the guidance and consent offered by community members. The ecosystem image (Figure 6) illustrates that preparation may include listening to stories of ongoing and historical harm, to process the related emotions, such as grief, and to unlearn as needed to arrive with an open mind. Overall, this theme highlights that entry should only be granted when the community feels connected to the researcher and the issue, with a readiness to be receptive and willing to engage in the cocreation and change. All cocreation participants should be invited to examine and identify their purpose, passion, and commitment to sustainable change from the outset of a project.

The idea of ‘going to community’ was strongly conveyed in images that showed a variety of physical locations, clearly highlighting that EqCC needs to occur where people are in their communities (e.g., homes, libraries, cafes, gardens, hairdressers), see Figures 1, 2, and 6. Researchers and community members must have the courage, energy, and motivation to move beyond traditional structures and systems to reach people who may not be accessing existing health or social services. The idea of finding and nurturing natural leaders within those spaces was shown in the sunflower (Figure 4) and the ecosystem (Figure 6). The people's social movement (Figure 2) provided the critical reminder to examine who is missing within those community spaces and to make purposeful efforts to include groups that are underrepresented.

All groups noted that EqCC begins with listening to the diverse stories that centre people's lived experiences and are foundational to building understanding and relationships. These stories generate emotion, allow for the identification of issues that people are passionate about, and prompt opportunities for iteration over time.

### 3.4 Theme 2: Nurture relationships and creativity

This second theme emphasises that effort is needed to create and nurture an optimal environment for creativity and relationship building. Colearning opportunities were prioritised in several of the images, noting the value of ample opportunities to observe and listen, build rapport, ask questions, and engage in experiential learning. This suggests that everyone must enter this space with an openness to learning and curiosity as shown in the soil of the sunflower (Figure 4) and value multiple ways of knowing as described in the house, the people's social movement, and the bottom left corner of the ecosystem (Figures 1, 2, and 6). The creation of relationships and a shared language allow people to share in diverse ways (e.g., storytelling, arts‐based methods). These relational means of colearning require trust and transparency and facilitate the creation of comfortable and safe spaces. Figure 3, disruptive pathways, reminds us that some spaces may instead aim towards vulnerable and brave spaces in which learning and unlearning can thrive via transformation and disruption. The house image (Figure 1) illustrates concrete strategies that can be used to enhance trust and transparency, such as introducing yourself and your purpose, asking questions to understand the cultural norms of the environment, and awaiting permission to be invited in.

The sunflower in Figure 4 most clearly illustrated the conditions needed for relationships and creativity to thrive in EqCC, shown as the *sun* which represented funding, and the *soil* that included time. These concepts were repeated in the disruptive pathways image (Figure 3) that identified time needed to change mindsets, build relationships and to build capacity. Funding in the form of salaries and investments needed for training and accreditation of community‐based leaders in EqCC was also illustrated in the new community ecosystem (Figure 6).

### 3.5 Theme 3: Reflect, replenish and grow

The new community ecosystem (Figure 6) most clearly illustrated the need for a ‘constant gardener’ as someone who could provide information about the ongoing and historical harms within a community; part of the preconditions required to reflect, replenish and grow. Before work begins, it was emphasised that cocreation participants should reflect, acknowledge harm, grieve, and be aware of the potential for introducing or reinforcing harms as shown in the house (Figure 1). The infinity loop (Figure 5) explains that cocreation processes can be exhausting, however the sunflower (Figure 3) showed that *rain* may flow via tears and stories to replenish the soil and create fertile ground for cocreation. The disruptive pathways (Figure 3) highlights that ongoing opportunities for feedback and reflection are needed, and the infinity loop (Figure 5) shows how this feedback allows teams to celebrate wins and serve as ‘energy’ boosters to sustain cocreation projects. Continuous reflection and feedback also provide opportunities for iteration, course correction, and to learn by doing. These continuous processes were illustrated in the infinity loop and disruptive pathways (Figures 5 and 3, respectively) that show initial stories that are transformed during cocreation to produce new stories that feedback into continued learning, growth, and progress. There should be opportunities to enter and exit this process as desired, as shown with the *open door* in the infinity loop (Figure 5). The new community ecosystem (Figure 6) provided a reminder that some *constant gardeners* are needed to remain over time to foster knowledge sharing.

### 3.6 Theme 4: Promote thriving and transformation

There are three elements that combine to create a vision for thriving and transformation, these include: consideration for who is engaged in the change, the processes by which thriving and transformation occur, and the impacts that can be achieved. The people's social movement (Figure 2) and the new community ecosystem (Figure 6) images most explicitly illustrated that change begins at the front line or grass root levels. People at the front line may enter EqCC by sharing their stories as illustrated in the *rain* in the sunflower (Figure 4), a *pathway* in the disruptive pathways (Figure 5) and the *book* in the infinity loop (Figure 4). These stories allow for the identification of new leaders who can then be supported in capacity building through training, recognition and resources (e.g., funding) as shown in the leaves of the sunflower (Figure 4) and the mesolayer of the new community ecosystem (Figure 6). Building from the ground up in this way creates a new workforce that promotes the redistribution of power and recognition of new leaders that can affect change at multiple levels of the ecosystem. These changes do not occur solely by opening doors for new people, they also require advocacy in challenging traditional systems and structures, illustrated in the sunflower as ‘protests to pressure government’ (Figure 4). Each win at the micro, meso, and macrolevels serves to build momentum, allowing for mutual reinforcement and a groundswell that promotes maximum impact. The impacts that were identified changed mindsets (Figure 3), policy change (Figure 4) and new forms of governance structures (Figure 6).

## 4 DISCUSSION

CoPro2022 was a series of events held as a 5‐year follow‐up to the inaugural CoPro2017 event held in Birmingham, UK where 28 participants (practitioners, academics, and service users) shared eight case examples over 2 days to ‘explore citizens' involvement in codesigning public services for vulnerable groups, identify challenges and suggest improvements’ to overcome barriers to their engagement.^9^ ^,p.1^ Key messages from the deliberations in 2017 were that power must be attended to throughout cocreation processes, for example, by being transparent in creating expectations, being flexible and responsive, and being accountable to the community. Relationships and trust were described as central in these processes and the CoPro2017 participants recommended that all cocreation participants adopt a stance of continuous learning and that service users should meaningfully inform cocreation approaches, tools, resources, and outcomes.

The CoPro2022 Forum expanded on the relationships built and lessons learned at CoPro2017 by increasing efforts to include EEs from EDGs in the event, including immersive and art‐based learning opportunities, and by identifying a collective vision for desired outcomes in cocreation. For example, from the perspective of increasing inclusivity and relationship‐building. CoPro2022 organisers were purposeful in their efforts made to include EEs from EDGs by paying for travel, accommodation and food, supporting language translation at the event, and inviting copresentations from people who lead cocreation projects in health and social services and lived EEs. Similarly, the venue selection was intentional; the event held at the Gathering Place by the Grand at Six Nations, the largest First nation in Canada, located along the banks of the Grand River in Ohsweken, Ontario to foster collaboration with the Six Nations of the Grand River Haudenosaunee people who hosted immersive learning opportunities, such as paddling, beading, and dancing. Art‐based activities fostered creative idea generation, discussion, and knowledge sharing (e.g., prototype development, graphic summaries of themes from the event, poetry with a dance presentation). These activities nurtured relationship building, allowing people to bring their full selves forward, and to experience both vulnerability and the building of trust. Event evaluation feedback was extremely positive, however, further evaluation would be required to determine if participants experienced lasting positive outcomes of engaging in cocreation as previously reported in the literature, such as increased skills, confidence, and a sense of accomplishment.^6^, ^19^ It is reported that codesigning services with EEs, service providers, leadership, policy makers, and researchers can lead to service improvements that better address the priorities of patients,^2^ however people from EDGs are often left out of these processes.^5^ Therefore, a vision for EqCC may aid in the design of cocreation projects that purposefully and meaningfully include people who are often marginalised. This may help to address the issue whereby cocreation processes and projects reproduce the exclusion of people from EDGs, resulting in services and systems that perpetuate marginalisation.

The vision for equity‐based cocreation established at CoPro2022 also considerably advances the field beyond what was heard at CoPro2017. As demonstrated in the visual prototypes developed in small groups, the collective vision expands beyond including marginalised groups in a given codesign or cocreation process, toward creating opportunities for thriving and transformation, by going to communities, adopting processes that enable deep ongoing relationship‐building and personal reflection, wherein community‐driven ecosystems can lead to transformative systems change over time, through the support of broader social movements for change. While each participant at CoPro2022 was able to share their own stories of cocreation objectives and positive outcomes achieved, there was an undeniable feeling of collective agency and a need to advance change together. Therefore, we have begun planning for the next CoPro event, with the inclusion of EEs at the planning table and a renewed and increased focus on creating a network led by and with EEs. Members of our team have submitted a grant to further understand the strengths in networks of EEs in mental health and childhood disability, while supporting these networks to increase their inclusion of people from EDGs. The community of participants and the momentum for change created at the event served as a microcosm for the kind of complex system change that was described as necessary by participants throughout the event discussions. While the importance of relationship and capacity building at an individual level was highlighted, there was agreement that these individual changes could build towards a network of change and redistribution in power and leadership. These changes may facilitate the advocacy needed to enable the systemic and pragmatic changes that are required to conduct sustainable and impactful cocreation (e.g., demonstrated commitment to co‐creation via grants that will compensate members of EDGs for their involvement, timelines that allow for relationship building, recognition for nonacademic outputs of cocreation work that benefit communities). This aligns with the redistribution of power in equity coproduction described by Akerlof et al.,^22^ such as diverse representation on the leadership team, compensation for those who participate, providing information and resources to people who participate, and ensuring the political leaders are involved. These broader changes in structures and policies were seen as the groundswell that could topple traditional hierarchies that exclude and exploit EDGs. Such change was viewed as necessary, aspirational, and feasible if people are committed, connected, skilled and informed in their efforts to advance a new collective vision for EqCC.

## AUTHOR CONTRIBUTIONS

**Michelle Phoenix**: Conceptualisation; investigation; funding acquisition; writing—original draft; methodology; validation; visualisation; writing—review and editing; formal analysis. **Sandra Moll**: Conceptualisation; investigation; funding acquisition; writing—review and editing; visualisation; methodology; validation; formal analysis. **Alexa Vrzovski**: Conceptualisation; investigation; validation; visualisation; methodology; writing—review and editing; formal analysis. **Le‐Tien Bhaskar**: Conceptualisation; investigation; validation; visualisation; writing—review and editing; formal analysis. **Samantha Micsinszki**: Conceptualisation; investigation; funding acquisition; validation; visualisation; writing—review and editing; formal analysis. **Emma Bruce**: Conceptualisation; investigation; funding acquisition; writing—review and editing; visualisation; validation. **Lulwama Mulalu**: Conceptualisation; visualisation; validation; writing—review and editing; formal analysis. **Puspita Hossain**: Conceptualisation; validation; visualisation; writing—review and editing; formal analysis. **Bonnie Freeman**: Validation; visualisation; writing—review and editing; investigation. **Gillian Mulvale**: Conceptualisation; investigation; funding acquisition; writing—original draft; methodology; validation; visualisation; writing—review and editing; formal analysis; project administration; supervision.

## CONFLICT OF INTEREST STATEMENT

The authors declare no conflict of interest.

## ETHICS STATEMENT

The CoPro2022 Forum was considered to be a knowledge exchange event and the studies presented had received the necessary ethical approvals from their host institutions. All participants signed a media release form giving permission to use the materials created at the CoPro2022 forum for further dissemination. The McMaster University Research Ethics Board did not require ethics approval for dissemination of the lessons learned at the CoPro2022 Forum.

## Data Availability

The refined versions of our original prototype images are available in the manuscript, these were the primary data source. Research data are not shared.

## References

[1] Nguyen T , Graham ID , Mrklas KJ , et al. How does integrated knowledge translation (IKT) compare to other collaborative research approaches to generating and translating knowledge? Learning from experts in the field. Health Res Policy Syst. 2020;18(1):35. 10.1186/s12961-020-0539-6 32228692 PMC7106699

[2] Bate P , Robert G . Bringing User Experience to Healthcare Improvement: The Concepts, Methods and Practices of Experience‐Based Design. Radcliffe Publishing; 2007.

[3] Moll S , Wyndham‐West M , Mulvale G , et al. Are you really doing ‘codesign’? Critical reflections when working with vulnerable populations. BMJ Open. 2020;10(11):e038339. 10.1136/bmjopen-2020-038339 PMC7640510 33148733

[4] Blomkamp E . The promise of co‐design for public policy. Aust J Public Adm. 2018;77(4):729‐743. 10.1111/1467-8500.12310

[5] Williams O , Sarre S , Papoulias SC , et al. Lost in the shadows: reflections on the dark side of co‐production. Health Res Policy Syst. 2020;18(1):43. 10.1186/s12961-020-00558-0 32380998 PMC7204208

[6] Slattery P , Saeri AK , Bragge P . Research co‐design in health: a rapid overview of reviews. Health Res Policy Syst. 2020;18(1):17. 10.1186/s12961-020-0528-9 32046728 PMC7014755

[7] Mulvale G , Miatello A , Green J , Tran M , Roussakis C , Mulvale A . A COMPASS for navigating relationships in co‐production processes involving vulnerable populations. Int J Public Adm. 2021;44(9):790‐802. 10.1080/01900692.2021.1903500

[8] Mulvale G , Green J , Miatello A , Cassidy AE , Martens T . Finding harmony within dissonance: engaging patients, family/caregivers and service providers in research to fundamentally restructure relationships through integrative dynamics. Health Expect. 2021;24(suppl 1):147‐160. 10.1111/hex.13063 32529748 PMC8137493

[9] Mulvale G , Moll S , Miatello A , et al. Codesigning health and other public services with vulnerable and disadvantaged populations: insights from an international collaboration. Health Expect. 2019;22(3):284‐297. 10.1111/hex.12864 30604580 PMC6543156

[10] Dixon‐Woods M , Cavers D , Agarwal S , et al. Conducting a critical interpretive synthesis of the literature on access to healthcare by vulnerable groups. BMC Med Res Methodol. 2006;6:35. 10.1186/1471-2288-6-35 16872487 PMC1559637

[11] Grabovschi C , Loignon C , Fortin M . Mapping the concept of vulnerability related to health care disparities: a scoping review. BMC Health Serv Res. 2013;13:94. 10.1186/1472-6963-13-94 23496838 PMC3626765

[12] Mulvale G , Moll S , Miatello A , Murray‐Leung L , Rogerson K , Sassi RB . Co‐designing services for youth with mental health issues: novel elicitation approaches. Int J Qual Methods. 2019;18. 10.1177/1609406918816244

[13] Micsinszki SK , Tanel NL , Kowal J , et al. Codesigning simulations and analyzing the process to ascertain principles of authentic and meaningful research engagement in childhood disability research. Res Involv Engagem. 2022;8(1):60. 10.1186/s40900-022-00398-y 36352487 PMC9645736

[14] Jamal S , Newbold KB . The promise of co‐design for improving transit service for older immigrants: development of a co‐design framework for Hamilton, Ontario. Urban Govern. 2023;3(1):83‐91. 10.1016/j.ugj.2023.01.002

[15] Terkelsen AS , Wester CT , Gulis G , Jespersen J , Andersen PT . Co‐creation and co‐production of health promoting activities addressing older people—a scoping review. Int J Environ Res Public Health. 2022;19(20):13043. 10.3390/ijerph192013043 36293629 PMC9602529

[16] Buchanan F . How do patients attain equal status if they're seen as ‘nonexpert’? Healthy Debate. 2019. https://healthydebate.ca/2019/03/topic/patients-equal-status/

[17] Gavin F . The risks of equating ‘lived experience’ with patient expertise. 2019. https://healthydebate.ca/2019/02/topic/patients-as-experts/

[18] Rowland P , Forest P‐G , Vanstone M , Leslie M , Abelson J . Exploring meanings of expert and expertise in patient engagement activities: a qualitative analysis of a pan‐Canadian survey. SSM Qual Res Health. 2023;4:100342. 10.1016/j.ssmqr.2023.100342

[19] Black A , Strain K , Wallsworth C , et al. What constitutes meaningful engagement for patients and families as partners on research teams? J Health Serv Res Policy. 2018;23(3):158‐167. 10.1177/1355819618762960 29504424 PMC6041763

[20] Gonzalez M , Phoenix M , Saxena S , et al. Strategies used to engage hard‐to‐reach populations in childhood disability research: a scoping review. Disabil Rehabil. 2021;43(19):2815‐2827. 10.1080/09638288.2020.1717649 31999495

[21] Pozniak K , Buchanan F , Cross A , et al. Building a culture of engagement at a research centre for childhood disability. Res Involv Engagem. 2021;7(1):78. 10.1186/s40900-021-00319-5 34742354 PMC8572501

[22] Akerlof KL , Timm KMF , Chase A , et al. What does equitable co‐production entail? Three perspectives. Commun Sci. 2023;2(2). 10.1029/2022csj000021 PMC12973253 41816204

[23] Oliver K , Kothari A , Mays N . The dark side of coproduction: do the costs outweigh the benefits for health research? Health Res Policy Syst. 2019;17(1):33. 10.1186/s12961-019-0432-3 30922339 PMC6437844

[24] McMaster University . CoPro2022 3‐Day International Forum. McMaster University Co‐Design VP Hub. 2023. https://codesign.mcmaster.ca/copro-2022/copro-2022-3-day-symposium/

[25] Mulvale G , Moll S , Phoenix M , et al. Co‐creating a new charter for equitable and inclusive co‐creation: insights from an international forum of academic and lived experience experts. BMJ Open. 2024;14(14):e078950. 10.1136/bmjopen-2023-078950 PMC10953044 38508634

[26] Micsinszki SK , Buettgen A , Mulvale G , et al. Creative processes in co‐designing a co‐design hub: towards system change in health and social services in collaboration with structurally vulnerable populations. Evid Pol. 2022;18(2):291‐310. 10.1332/174426421x16366319768599

[27] Mulvale A , Miatello A , Hackett C , Mulvale G . Applying experience‐based co‐design with vulnerable populations: lessons from a systematic review of methods to involve patients, families and service providers in child and youth mental health service improvement. Patient Exp J. 2016;3(1):117‐129. 10.35680/2372-0247.1104

[28] Sanders EBN , Stappers PJ . Probes, toolkits and prototypes: three approaches to making in codesigning. CoDesign. 2014;10(1):5‐14. 10.1080/15710882.2014.888183

